# Improving Human Happiness Analysis Based on Transfer Learning: Algorithm Development and Validation

**DOI:** 10.2196/28292

**Published:** 2021-08-06

**Authors:** Lele Yu, Shaowu Zhang, Yijia Zhang, Hongfei Lin

**Affiliations:** 1 College of Computer Science and Technology Dalian University of Technology Dalian China

**Keywords:** happiness analysis, sentiment analysis, transfer learning, text classification

## Abstract

**Background:**

Happiness refers to the joyful and pleasant emotions that humans produce subjectively. It is the positive part of emotions, and it affects the quality of human life. Therefore, understanding human happiness is a meaningful task in sentiment analysis.

**Objective:**

We mainly discuss 2 facets (Agency/Sociality) of happiness in this paper. Through analysis and research on happiness, we can expand on new concepts that define happiness and enrich our understanding of emotions.

**Methods:**

This paper treated each happy moment as a sequence of short sentences, then proposed a short happiness detection model based on transfer learning to analyze the Agency and Sociality aspects of happiness. First, we utilized the unlabeled training set to retrain the pretraining language model Bidirectional Encoder Representations from Transformers (BERT) and got a semantically enhanced language model happyBERT in the target domain. Then, we got several single text classification models by fine-tuning BERT and happyBERT. Finally, an improved voting strategy was proposed to integrate multiple single models, and “pseudo data” were introduced to retrain the combined models.

**Results:**

The proposed approach was evaluated on the public dataset happyDB. Experimental results showed that our approach significantly outperforms the baselines. When predicting the Agency aspect of happiness, our approach achieved an accuracy of 0.8653 and an F1 score of 0.9126. When predicting Sociality, our approach achieved an accuracy of 0.9367 and an F1 score of 0.9491.

**Conclusions:**

By evaluating the dataset, the comparison results demonstrated the effectiveness of our approach for happiness analysis. Experimental results confirmed that our method achieved state-of-the-art performance and transfer learning effectively improved happiness analysis.

## Introduction

As the pressure of social life increases, people’s mental health has also received extensive attention. Taking depression as an example, the World Health Organization reported that more than 350 million people suffer from depression, and the growth in the rate of patients with depression over the past 10 years is about 18%. From these data, psychological illness has an essential impact on human health and has become the leading cause of health problems. Therefore, sentiment analysis has become a valuable research hotspot. Happiness is a positive part of the sentiment, and research on happiness also has the prospect of practical application and the value of sentiment analysis.

**Figure 1:**
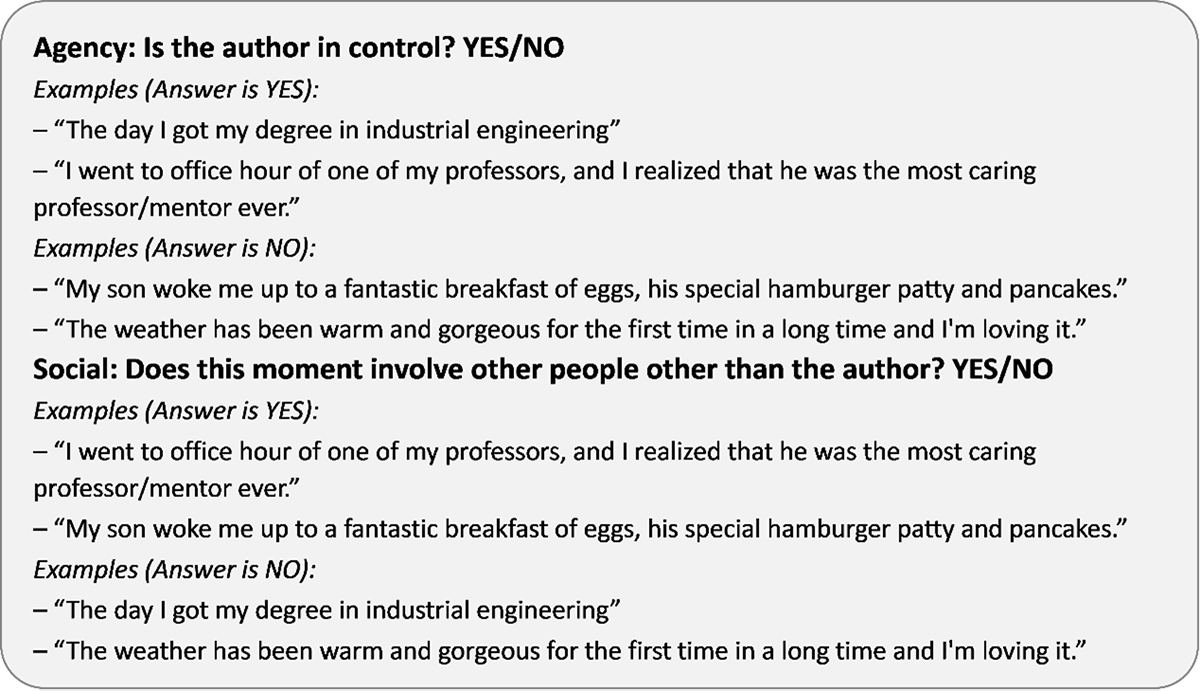
Examples of happy moments along two binary dimensions: Agency and Sociality.

The current research on happiness mainly comes from the CL-Aff Shared Task 2019: in Pursuit of Happiness [1]. This shared task has published 2 tasks. The first task is a semisupervised classification task: predict thematic labels (Agency and Sociality) on unseen data, based on small labeled and large unlabeled training data. The second task is to suggest interesting ways to automatically characterize the happy moments in terms of affect, emotion, participants, and content. Our focus is on the first task, and we challenge the current understanding of emotion through a task that models the experiential, contextual, and agentic attributes of happy moments. This paper mainly explores 2 aspects of happiness, namely Agency and Sociality. Agency mainly focuses on whether happy moments are dominated by people, while Sociality focuses more on whether happy moments involve other people. As shown in Figure 1, from the sentence “The day I got my degree in industrial engineering,” we can see that this happy moment comes from the author's degree and the author controls this behavior. Therefore, the Agency label for this happy moment is set to “YES”; at the same time, this happy moment does not involve other people, so the Sociality label of this happy moment corresponds to “NO.” It can be seen from this example that our proposed method should focus on different aspects of sentences. Therefore, we used inconsistent text classification models to predict the Agency and Sociality of happiness.

Happiness analysis is an essential part of sentiment analysis, which aims to classify the Agency and Sociality of a happy moment and be regarded as a typical text classification task. Traditional text classification methods are mainly based on machine learning methods, such as feature engineering. For feature engineering, the most commonly used feature is the bag-of-words feature. In addition, some more complex features have been proposed, such as n-grams [2] and entities in ontologies [3]. These methods have achieved good results in text classification tasks, but they require much manual intervention and consume a lot of time and energy. Recently, deep learning technology has gradually replaced traditional machine learning technology as the mainstream method for text classification [4]. For example, Mikolov et al [5] proposed the neural network–based language models Continuous Bag of Words (CBOW) and Skip-gram as well as distributed word vectors. Kim [6] proposed a multiscale, parallel, single-layer convolutional neural network (CNN) combined with pretrained word vectors to achieve sentence-level text classification. Hochreiter and Schmidhuber [7] proposed long short-term memory (LSTM) for text classification to solve the problem of gradient disappearance and gradient explosion in the original recurrent neural network (RNN) during training. Vaswani et al [8] proposed a transformer mechanism in which the encoder and decoder are formed by stacking the basic feedforward neural network and attention mechanism. The aforementioned methods play an important role in text classification tasks in a field, but there are some limitations in short text classification tasks for detecting happiness. The main reasons are that the size of the dataset is small, the text length of the dataset is short, the context of sentences is not close, and the number of emotional words contained in the text of the dataset is too small. Therefore, we proposed a method based on transfer learning and deep learning to solve these problems.

With the emergence of more machine learning application scenarios, the existing better-performing supervised learning requires a large amount of labeled data. However, labeling data is a tedious and costly task, so transfer learning has received increasing attention. Transfer learning has significant influence in the field of computer vision. Most models applied in the computer vision field use existing models for fine-tuning and rarely train from scratch. Pretrained models are obtained on big data such as ImageNet and MS-COCO [9-11]. The transfer learning currently applied to natural language processing (NLP) is mainly aimed at the first layer of the model. By fine-tuning the pretrained word embedding, it can be considered a simple transfer learning technique, but it has great value in practical applications and can be applied to various deep learning models. Based on transfer learning, we used model fine-tuning to complete the task of short text classification about happiness. To improve model performance and training efficiency, we used the triangle learning rate [12] and made full use of the hidden layer state information of the model. At the same time, transfer learning has also been widely applied to NLP. Embeddings from Language Models (ELMo) [13] appeared as a dynamic word vector in 2018, expressing different words in different contexts. Devlin et al [14] and others proposed a pretraining language model called Bidirectional Encoder Representations from Transformers (BERT) in 2018, which adopted a general pretraining model for more extensive and more profound network training.

This paper treated the happiness analysis task as a short text classification task and implemented transfer learning based on BERT. Considering the effectiveness of the pretrained model, we used model-tuned transfer learning technology to complete the task of happiness analysis. The main contributions of this paper are as follows. First, we got a semantic enhancement model happyBERT in the target domain by retraining BERT. The experimental results confirmed that domain-specific BERT outperforms general domain BERT on the HappyDB dataset [15]. Second, by fine-tuning the classification model, we mainly compared the influence of [CLS] tokens in different hidden layers of the model and the influence of other tokens in the last hidden layer on the experimental results and the further combination of the model and the deep learning neural network. The experiment proved that the fine-tuned model improved experimental results. We merged the fine-tuned model. Then, we proposed an improved voting fusion strategy to fuse the fine-tuning model, which could get the best model fusion combination, and introduced the “pseudo data” to retrain the model combination. Third, the experimental results showed that our proposed model achieved state-of-the-art performance in the task of happiness analysis.

## Methods

### Architecture

**Figure 2:**
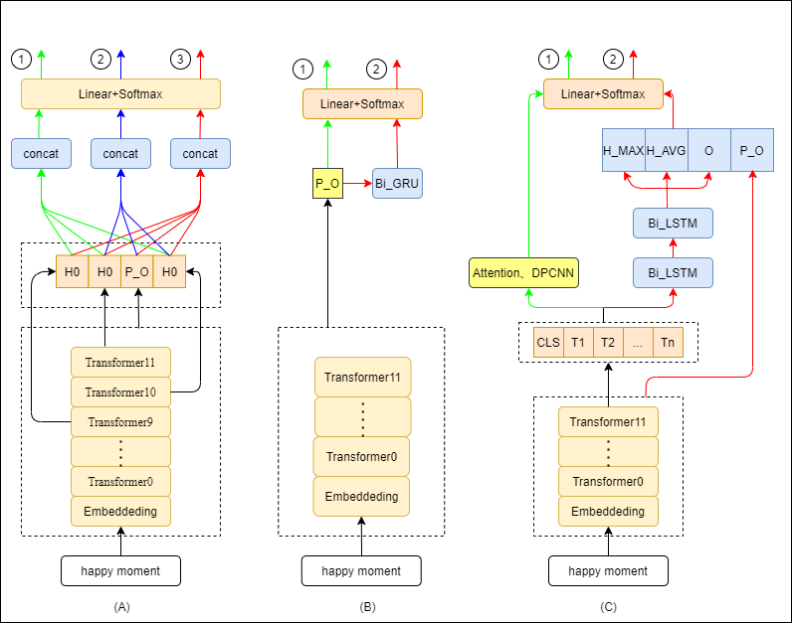
Introduction to the model structure used in the experiment: (A) extract the first state output of the hidden layer inside the model, (B) extract the model pooler_output, and (C) utilize all the state information of the last hidden layer of the model. BiLSTM: bidirectional long short-term memory; DPCNN: deep pyramid convolutional neural networks.

Our proposed model architectures (Figure 2) take as input preprocessed data (data splicing, data cleaning), which is input into the pretraining language model at a word level, and output “YES” or “NO” over a discrete label space. Unlike the general methods, we focus on the [CLS] token of the last layer of the language model and focus on the other tokens in the last layer of the language model and the output of other layers. We spliced these outputs with neural network models and got the classification results through the softmax layer. The pooler_output represents the hidden state of the first token of the sequence further processed by linear layer and Tanh activation function in the last layer of BERT or happyBERT. Based on the BERT model and happyBERT model, we made the following improvements. We extracted the first state output of the hidden layer in the model (Figure 2A). Then, we concatenated the first status output of the last 3 layers and passed a fully connected layer to achieve classification, as shown in 1. We concatenated the pooler_output and the first status output of the last 2 layers, then passed a fully connected layer to achieve classification, as shown in 2. Finally, we concatenated the pooler_output and the first status output of the last 3 layers, then passed a fully connected layer to achieve classification, as shown in 3.

As shown in Figure 2B, we extracted the model pooler_output and directly used the pooler_output of the original model for classification, which is also the common method of the original model for classification, as shown in 1. Then, we used the pooler_output of the original model as the input of the upper BiGRU [16] and then classified as shown in 2.

As shown in Figure 2C, we utilized all the state information of the last hidden layer of the model. All the last hidden layer state information can be used as input and then connected to other network models, such as self-attention and deep pyramid convolutional neural networks (DPCNN) [17]. Then, we classified it, as shown in 1. The status information can be connected to deeper network models, such as bidirectional LSTM (BiLSTM) and bidirectional gated recurrent unit (BiGRU) [16]. We extracted the higher-dimensional features of the text through a deeper network model and then aggregated the BiGRU output and hidden layer state features by extracting the hidden layer state, average pooling, and max pooling, finally concatenating the pooler_output of the BERT model for classification, as shown in 2.

The research was mainly divided into 3 stages: The first stage was fine-tuning the pretrained language model BERT, the second stage was to transform the upper structure of the language model obtained in the first stage to obtain a text classification model and then fine-tune the classification model, and the third stage was to ensemble the classification model obtained in the second stage, so we could get the best model combination, and then introduce “pseudo data” to retrain the best combination models to improve the overall classification results.

### Language Model

Observing the overall architecture of the model, there are many deep learning models used in this architecture. The following sections mainly introduce the language models.

#### BERT

We chose the pretraining language model BERT in this study. Proposed by the Google AI research institute in October 2018, BERT is a pretraining model that can achieve excellent machine reading comprehension, text classification, and other NLP tasks. This study adopted the base version of BERT, which is named BERT_base. BERT_base has less parameter information compared with BERT_large. On the BERT_based, the number of Transformer blocks is 12, the hidden layer size is 768, the number of self-attention heads is 12, and the total number of parameters for the pretrained model is 110,000,000.

#### happyBERT

The general field dataset used by Google to train the BERT model is very diverse, but the data in the relative happiness field have different distributions. Since the HappyDB dataset [15] contains a large amount of unlabeled data, we retrained BERT on the unlabeled corpus and updated the weights of the original BERT. Then, the resulting new pretraining model was called happyBERT. To adapt the pretrained language model to the happiness analysis task, we fine-tuned the model using the tilted triangular learning rate to quickly converge to the appropriate region of the parameter space at the beginning of training and optimize its parameters.

#### BiLSTM

LSTM is an improved RNN model based on RNN, which is widely used in many NLP tasks. The LSTM model overcomes the vanishing gradient problem by introducing a gating mechanism. Therefore, it is suitable to capture the long-term dependency feature. The LSTM unit consists of 3 components: the input gate *i_t_*, the forget gate *f_t_*, and the output gate *o_t_*. At the time step *t*, the LSTM unit utilizes the input word *x_t_*, the previously hidden state *h*_(_*_t_*_–1)_ and the previous cell state *c*_(_*_t_*_–1)_ to calculate the currently hidden state *h_t_* and cell state *c_t_*. The equations are as follows:

*f_t_* = σ(*W_f_ x_t_* + *U_f_ h*_(_*_t_*_–1)_ + *b_f_*) **(1)**

*o_t_* = σ(*W_o_ x_t_* + *U_o_ h*_(_*_t_*_–1)_ + *b_o_*) **(2)**

g*_t_* = σ(*W_g_ x_t_* + *U_g_ h*_(_*_t_*_–1)_ + *b_g_*) **(3)**

*i_t_* = σ(*W_i_ x_t_* + *U_i_ h*_(_*_t_*_–1)_ + *b_i_*) **(4)**

*c_t_* = *f_t_*
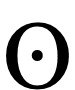
*c*_(_*_t_*_–1)_ + *i_t_*
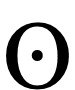
g*_t_***(5)**

*h_t_* = *o_t_*
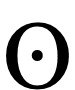
tanh (*c_t_*) **(6)**

where W, U, b are the weight and bias parameters and 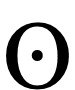 denotes element-wise multiplication. This study uses the BiLSTM model that can simultaneously capture the forward and backward context features. The BiLSTM model combines a forward LSTM and a backward LSTM.

#### BiGRU

GRU can be regarded as a variant of LSTM. GRU replaces the forget gate and the input gate in LSTM with the update gate *z_t_*. Combining the cell state and the hidden state *h_t_*, calculating the new information at the current moment is different from that with LSTM. The following figures show the process of GRU updating *h_t_*:

*r_t_* = σ(*W_r_ x_t_* + *U_r_ h*_(_*_t_*_–1)_ + *b_r_*) **(7)**

*z_t_* = σ(*W_z_ x_t_* + *U_z_ h*_(_*_t_*_–1)_ + *b_z_*) **(8)**

*h_t_* = tanh(*W x_t_* + *r_t_ Uh*_(_*_t_*_–1)_ + *b*) **(9)**

*h_t_* = (1 – *z_t_*) + *z_t_ h*_(_*_t_*_–1)_) **(10)**

where W, U, b are the weight and bias parameters. The BiGRU model combines a forward GRU and backward GRU.

#### Self-Attention

Attention was first proposed in 2017, and self-attention is one of the mechanisms. Different from general Attention, self-attention is the Attention of the sentence itself. To calculate self-attention, we need to declare the 3 vectors *Q*, *K*, and *V*. These vectors are obtained by dot multiplication of the word embedding vector H and the training matrix W created in the training process, including *Q* = *HW^Q^*, *K* = *HW^K^*, and *V* = *HW^V^*. The formula for calculating Attention is as follows:

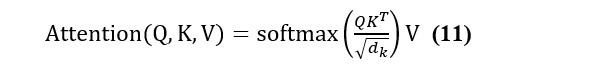



where *Q*, *K*, and *V* 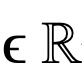
*^n^* represent the 3 matrices of query, key, and value, respectively, and *d* represents the dimension of *K*.

#### DPCNN

The DPCNN [17] model was first proposed in 2017. The model belongs to a low-complexity, word-level, deep CNN text classification architecture. By continuously deepening the network, it can solve the problem that the traditional CNN model cannot obtain the long-distance dependence of the text through convolution, so it can effectively represent the long-distance dependence of the text. With the deepening of the deep learning network, the related computational complexity also increases, bringing severe challenges to practical application. The DPCNN model is based on the deepening of word-level CNN to obtain the global representation of the text. The best accuracy can be obtained by increasing the network depth without increasing computational cost by much.

### Classification Model

For the happiness analysis, we first retrained BERT to get the happyBERT model. Second, we made many attempts on the model output and used 4 different deep learning models to achieve classification. The deep learning models include DPCNN, BiLSTM, BiGRU, and self-attention; the model classifiers formed by splicing them with the aforementioned BERT and happyBERT models are as follows: bert_last3embeddingcls, happybert_last3embeddingcls, bert_last2embeddingcls, happybert_last2embeddingcls, bert_last3embedding,happybert_last3embedding, bert_base, happybert_base, bert+attention, happybert+attention, bert+gru, happybert+gru, bert+grulstm, happybert+grulstm, bert+dpcnn, happybert+dpcnn. In these, the “+” means that the output of the last transformer layer of the pretraining model is input to the corresponding layer of the classification model, “_” means that the output of the pretraining model is adjusted, and the last3embedding represents the 1 in Figure 2A. The last2embeddingcls and last3embeddingcls represent 2 and 3 in Figure 2A. The base represents the pooler_output of the pretraining model. To get the result, the input of a fully connected layer is classified directly.

### Model Ensemble and “Pseudo Data”

We thought about improving the single model in general tasks at first, but when the single model encountered a bottleneck, we utilized a model ensemble to improve the experimental results further. There are many methods for a model ensemble; we used a voting mechanism to improve the performance of the entire classification system.

Through the analysis of happy moments via BertViz [18], different models pay extra attention to happy moments. Therefore, different model combinations have different voting results on Agency and Society. When predicting Agency, the best model combination was happybert_last3embedding, happybert_base, bert+grulstm, bert+attention, bert_base, and voting between these 5 models; the best results can be obtained on the validation set. We used the voting results of the obtained 5 models on the test set as the final classification result. Accuracy reached 0.8574, and the F1 score reached 0.9000. Furthermore, when predicting Sociality, the best model combination was happybert+attention, happybert_last3embeddingcls, happybert+grulstm, bert+dpcnn, happybert+dpcnn, happybert+gru, bert_base, happybert_base, and voting between these 8 models. The results can achieve the best performance on the validation set, and then the voting results of the 8 models on the test set were used as the final classification result. The accuracy reached 0.9280, and the F1 score reached 0.9360. This paper used the best_com_voting model to represent the model combination that achieves the best results on the validation set.

Since the HappyDB dataset has many unlabeled training sets, it is worth paying attention to accurately using this part of the data in the experiment. In this study, we used the unlabeled training set as the test set of the single model in the aforementioned optimal model combination, and each unlabeled training set obtained the prediction results; we added these training set data as “pseudo data” into the original labeled training set and then retrained the models in the optimal model combination. Finally, these newly obtained models were used to obtain the prediction results on the test set through a voting strategy. We used the best_com_pse model to represent these newly obtained model combinations. When predicting the Agency aspect of happiness, we achieved an accuracy of 0.8653 and an F1 score of 0.9126. When predicting Sociality, we achieved an accuracy of 0.9367 and an F1 score of 0.9491.

## Results

### Dataset and Task Description

The happiness analysis task based on transfer learning originates from the CL-Aff Happiness Shared Task 1. According to the predefined happy moment given by the official, it returns “YES” or “NO” in the Agency and Sociality dimensions. The HappyDB dataset used in this paper is from the CL-Aff Happiness Shared Task, which includes a labeled training set, unlabeled training set, and test set. The statistics for the number of datasets are shown in Table 1.

**Table 1:** Statistics of the HappyDB dataset.

Dataset	Agency		Sociality		Total, n
	Positive, n	Negative, n	Positive, n	Negative, n	
Labeled training set	7796	2764	5625	4935	10,560
Unlabeled training set	-^a^	-^a^	-^a^	-^a^	72,324
Test set	12,156	5059	9798	7417	17,215

^a^Not applicable.

### Assessment Criteria

We evaluated the performance of the happiness analysis task by using the F1 score and accuracy, as follows:

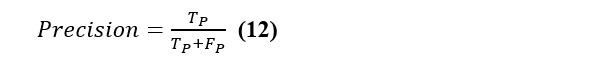

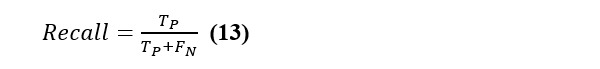

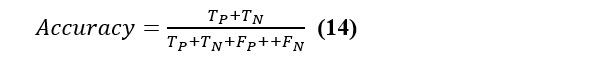

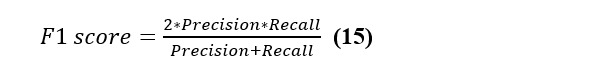









where *T_p_* represents true positive, *F_p_* represents false positive, *T_n_* represents true negative, and *F_n_* represents false negative.

### Experiment Settings

#### Hyperparameter Settings

The model codes used in this task were modified and implemented based on the open-source project transformers of the HunggingFace team [19]. The pretraining language model used was the BERT pretraining model provided by the Google team. To save memory, a single GPU batch size during fine-tune was set to 4; gradient accumulation steps were set to 4. Hence, every time 1 sample was input, the gradient was accumulated 4 times, and then backpropagation was performed to update the parameters to sacrifice a certain training speed. The hyperparameter settings used in the experiment are shown in Table 2. The dropout rate of the model was set to 0.1, and the learning rate was set to 1e-5. Since the HappyDB dataset belongs to the short text dataset, the sequence length was set to 56. In addition, the number of training steps and some parameters of DPCNN and LSTM were set.

**Table 2:** Hyperparameter settings.

Parameter	Value	Parameter	Value
Dropout rate	0.1	Filter num (DPCNN^a^)	256
Learning rate	1e-5	Filter size (DPCNN)	3
Max sequence length	56	Block size (DPCNN)	2
Optimizer	AdamW	Hidden size (LSTM^b^)	128
Training steps	30,000	Bidirectional (LSTM)	True

^a^DPCNN: deep pyramid convolutional neural network.

^b^LSTM: long short-term memory.

#### Loss Function

Since the happiness task involves 2 subtasks, which are Agency and Sociality classifications of the Happy moment, these 2 subtasks contained 2 categories (Agency: “YES” and “NO”; Sociality: “YES” and “NO”). These 2 subtask sample categories were relatively balanced and easy to distinguish. We used the standard cross-entropy loss function as the loss function of the happiness task:

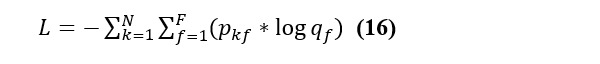



where N is the number of samples and *F* is the dimension of the output feature*,* which is equal to the number of classes. And, *p* is the true value, and *q* is the predicted value after softmax.

### Our Methods and Analysis

We finally implemented 16 neural network models for happiness detection. For each model, we adopted a 5-fold cross-validation of stratified sampling. Stratified sampling ensured that the proportion of samples in each category in each fold dataset remained unchanged. The model with the highest F1 score on the validation set was selected to predict the test set, and the probability average was used for the final 5-fold fusion. Then, we used voting to do the final model fusion of these models and selected the best model combination. Finally, we introduced “pseudo data” to retrain the single model in the best combination model so that a new single model could be obtained, and then, these new models could be fused by a voting strategy. The classification results for Agency and Sociality are shown in Table 3 and Table 4.

As we can see from Table 3 and Table 4, when predicting Sociality, the happybert+dpcnn model achieved the best result of the 12 single models, with an F1 score of 0.9350; thus, it can be proved that after the language model, a splicer neural network model can improve the classification results on specific tasks. Fine-tuning the model can improve the classification results. When predicting Agency, the happybert_last3embeddingcls model achieved the best results; the F1 score was 0.8987. Different pretraining models and different deep learning neural network models can be spliced to obtain different experimental results. The knowledge characteristics learned from the HappyDB dataset [15] for every single model were different. The integrated models can complement each other to improve the performance of the entire classification system. In addition, adding “pseudo data” to the training set can expand the scale of the dataset, thus effectively improving the performance of the classification system. For predicting Agency, the F1 score we finally submitted was 0.9126, and the accuracy was 0.8653; the F1 score was 1.57% higher, and the accuracy was 1.1% higher than bert_base. For predicting Sociality, the F1 score was 0.9421, the accuracy was 0.9367; the F1 score was 1.62% higher, and the accuracy was 1.18% higher than bert_base, proving the effectiveness of our model.

**Table 3:** Experimental results for Agency and Sociality.

Models	Agency		Sociality	
	Accuracy	F1	Accuracy	F1
bert_base	0.8543	0.8969	0.9249	0.9332
happybert_base	0.8545	0.8959	0.9264	0.9347
bert+attention	0.8515	0.8955	0.9247	0.9330
happybert+attention	0.8516	0.8943	0.9244	0.9324
bert+grulstm	0.8531	0.8983	0.9203	0.9289
happybert+grulstm	0.8491	0.8968	0.9197	0.9289
bert_last2embeddingcls	0.8512	0.8980	0.9157	0.9291
happybert_last2embeddingcls	0.8530	0.8982	0.9197	0.9289
bert_last3embedding	0.8516	0.8955	0.9159	0.9278
happybert_last3embedding	0.8528	0.8986	0.9189	0.9305
bert+gru	0.8497	0.8964	0.9255	0.9335
happybert+gru	0.8532	0.8969	0.9260	0.9340
bert+dpcnn	0.8514	0.8948	0.9253	0.9332
happybert+dpcnn	0.8567	0.8958	0.9268	0.9350
bert_last3embeddingcls	0.8522	0.8978	0.9200	0.9285
happybert_last3embeddingcls	0.8536	0.8987	0.9180	0.9272
all_voting	0.8554	0.8997	0.9268	0.9349
best_com_voting	0.8574	0.9000	0.9280	0.9360
best_com_pse	0.8653	0.9126	0.9367	0.9491

**Table 4:** Results of the ablation experiments for Agency and Sociality.

Models	Agency		Sociality	
	Accuracy	F1	Accuracy	F1
bert	0.8489	0.8902	0.9154	0.9301
bert_fine	0.8543	0.8969	0.9249	0.9332
bert_best_com	0.8551	0.8996	0.9268	0.9347
bert_com_pse	0.8623	0.9086	0.9293	0.9417

### Ablation Study

In order to verify the effectiveness of fine-tuning strategies, model fusion strategies, and the introduction of “pseudo data,” we set up ablation experiments for comparison. The results are shown in Table 4, where bert_fine means fine-tuning the pretraining language model BERT.Compared with bert without fine-tuning, when predicting Agency, fine-tuning the language model can improve accuracy by 0.54% and the F1 score by 0.67%. When predicting Sociality, fine-tuning the language model can improve the accuracy by 0.95% and the F1 score by 0.31%, which fully proves the effectiveness of the fine-tuning model. The bert_best_com model represents the best model voting combination based on the BERT model. Compared with bert_fine, the bert_best_com model can improve the accuracy by 0.08% and the F1 score by 0.15% when predicting Agency and can increase the accuracy by 0.19% and the F1 score by 0.15% when predicting Sociality, which fully proves the effectiveness of model fusion. bert_com_pse represents the model combination obtained by introducing “pseudo data” based on the bert_best_com model. When bert_com_pse predicts Agency, it can increase the accuracy by 0.72% and the F1 score by 0.90%. When predicting Sociality, it can increase the accuracy by 0.25% and the F1 score by 0.70%, which fully proves the effectiveness of introducing “pseudo data.”

### Compared Experiments and Analysis

We used the following classification models to conduct comparative experiments on the HappyDB dataset to verify the effectiveness of the proposed model. For IoH-RCNN, we utilized a recurrent convolutional neural network (RCNN) and combined words with their context to get a more precise word embedding. For SAWD-LSTM, we employed an inductive transfer learning technique, pretrained an AWD-LSTM neural net on the WikiText103 corpus, and then introduced an extra step to adapt the model to happy moments. For XGBoosted Forest and CNN, we used different feature sets to train their model, including syntactic features, emotional features, and survey features. Then, we used semisupervised learning and experimented with XGBoosted Forest and CNN models.

The results of the comparative experiment are shown in Table 5. It can be seen that our proposed method achieves the best results on the HappyDB dataset, verifying the effectiveness of transfer learning on the task of happiness analysis.

**Table 5:** Experimental results of the existing methods.

Models	Agency			Sociality	
	Accuracy	F1	Accuracy		F1
IoH-RCNN^a^	0.83	0.89	0.91		0.92
SAWD-LSTM^b^	0.84	0.89	0.92		0.93
XGBoosted Forest and CNN	0.83	0.88	0.89		0.90
best_com_pse (our model)	0.86	0.91	0.93		0.94

^a^RCNN: recurrent convolutional neural network.

^b^LSTM: long short-term memory.

### Error Analysis

To understand our model better, we performed error analyses on the output of our final results. We observed that in some of the cases (eg, “When I got my first paycheck”), the bert_base model predicted Sociality “YES” but the happybert_base model predicted Sociality “NO”; in fact, when Sociality is “NO,” the happy_bert model learned more on the Sociality classification. When predicting “I was happy to hear from my sister,” the bert_base model predicted Agency “NO,” but the bert_last3embedding model predicted agency “YES”; in fact, when the Agency is “NO,” the bert_last3embedding model performed better on Agency classification. In the future, we will consider preferable preprocessing and postprocessing techniques to solve these problems.

### Visualization of Attention Maps in BERT

**Figure 3:**
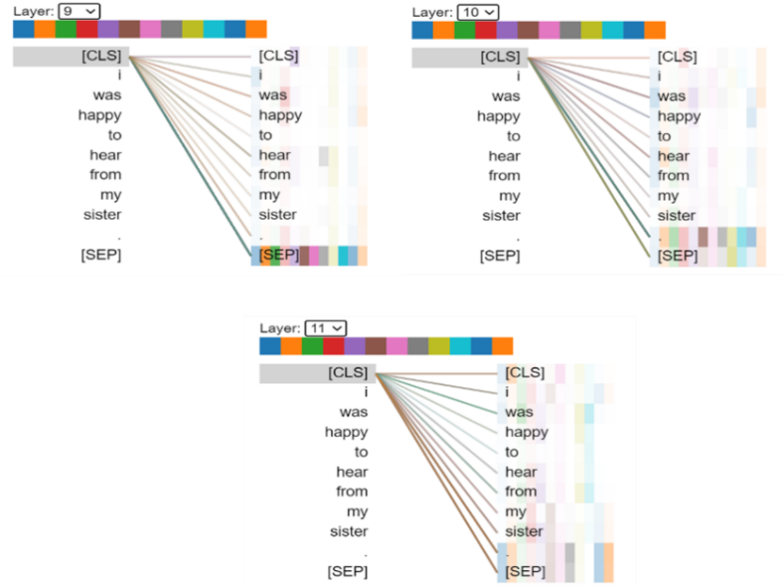
Visualization of different layer attention in an example sentence via BertViz [19].

Visualization can help us understand how BERT forms representations of text to understand languages. Figure 3 reveals the last 3 layers’ attention induced by a sample input text. We can see that the [CLS] of the last 3 layers of BERT had inconsistent attention to the same word, which is consistent with our proposed model concept. Our model combined the output of multiple Transformer layers of BERT to form the final output. Such attention information helped predict Agency and improved our model performance.

## Discussion

This paper proposed happyBERT. The happyBERT model is obtained by retraining BERT using an unlabeled training corpus in the HappyDB dataset. The purpose of retraining is to update the BERT parameters. Compared with BERT, happyBERT is more domain-relevant so that it can show better results on happiness analysis tasks, and the experimental results can better support this.

The contributions of different layers of BERT and different tokens of the same layer to the task were inconsistent. In the experimental section, we discussed the impact of the token in the BERT’s last 3-layer Transformer on the experiment. Based on this thinking, we proposed single models based on BERT and happyBERT. The classification results of every single model on Agency and Sociality are given. In subsequent experiments, we also introduced an improved model fusion strategy and “pseudo labels.” These strategies also improved the performance of the classification model to a certain extent.

### Limitations

The happiness analysis is a novel task. So far, HappyDB is the only public dataset in this field. Moreover, only about 10,000 of the data in HappyDB are labeled. One of the limitations is that our method was only evaluated on HappyDB. In future work, we plan to annotate a larger dataset for happiness analysis.

Another limitation of our study is that we only evaluated the effectiveness of the BERT model. In recent studies, the latest pretrained models, such as Roberta [20] and GPT [21], have successfully applied NLP tasks. In a future study, we will validate these latest pretrained models on the happiness analysis task.

### Conclusion

We proposed a happiness detection model based on transfer learning. Our approach utilized an unlabeled training set for training a semantically enhanced language model in the target domain and fine-tune the language model. Model fusion was applied to improve the performance of the entire happiness detection system. In addition, “pseudo data” were also introduced, which can further improve the classification performance. The experimental results suggest that our method achieves state-of-the-art performance, fully demonstrating the effectiveness of our method.

## Abbreviations

BERT: Bidirectional Encoder Representations from Transformers
BiGRU: bidirectional gated recurrent unit
BiLSTM: bidirectional long short-term memory
CBOW: Continuous Bag of Words
CNN: convolutional neural network
DPCNN: deep pyramid convolutional neural networks
ELMo: Embeddings from Language Models
GRU: gated recurrent unit
LSTM: long short-term memory
NLP: natural language processing;
RCNN: recurrent convolutional neural network
RNN: recurrent neural network
